# HnRNP F/H associate with hTERC and telomerase holoenzyme to modulate telomerase function and promote cell proliferation

**DOI:** 10.1038/s41418-019-0483-6

**Published:** 2019-12-20

**Authors:** Chenzhong Xu, Nan Xie, Yuanyuan Su, Zhaomeng Sun, Yao Liang, Na Zhang, Doudou Liu, Shuqin Jia, Xiaofang Xing, Limin Han, Guodong Li, Tanjun Tong, Jun Chen

**Affiliations:** 1 Peking University Research Center on Aging, Beijing Key Laboratory of Protein Posttranslational Modifications and Cell Function, Department of Biochemistry and Molecular Biology, Department of Integration of Chinese and Western Medicine, School of Basic Medical Science, Peking University, Beijing, 100191 China; 2 Department of Physiology and Pathophysiology, School of Basic Medical Science, Peking University, Beijing, 100191 China; 3 Department of Molecular Diagnostics, Key Laboratory of Carcinogenesis and Translational Research (Ministry of Education), Peking University Cancer Hospital & Institute, Beijing, 100142 China

**Keywords:** RNA-binding proteins, RNA

## Abstract

Human telomerase RNA component hTERC comprises multiple motifs that contribute to hTERC biogenesis, holoenzyme activity, and enzyme recruitment to telomeres. hTERC contains several guanine tracts (G-tracts) at its 5′-end, but its associated proteins and potential roles in telomerase function are still poorly understood. The heterogeneous nuclear ribonucleoproteins F, H1, and H2 (hnRNP F/H) are splicing factors that preferentially bind to poly(G)-rich sequences RNA. Here, we demonstrate that hnRNP F/H associate with both hTERC and telomerase holoenzyme to regulate telomerase activity. We reveal hnRNP F/H bind to the 5′-end region of hTERC in vitro and in vivo, and identify the first three G-tracts of hTERC and qRRM1 domain of hnRNP F/H are required for their interaction. Furthermore, hnRNP F/H also directly interact with telomerase holoenzyme. Functionally, we show that hnRNP F/H plays important roles in modulating telomerase activity and telomere length. Moreover, hnRNP F/H deletion greatly impair cancer and stem cell proliferation, and induce stem cell senescence, while hnRNP F/H overexpression delay stem cell senescence. Collectively, our findings unveil a novel role of hnRNP F/H as the binding partners of hTERC and telomerase holoenzyme to regulate telomerase function.

## Introduction

Telomerase is a specialized reverse transcriptase ribonucleoprotein (RNP) complex composed of two major components, a catalytic telomerase reverse transcriptase (hTERT) protein and a noncoding RNA component (human telomerase RNA component, hTERC). hTERC serves as a template to provide the template sequence for hTERT to catalyze the addition of telomeric DNA repeats to the chromosome ends to maintain telomere length. Both hTERT and hTERC are required for telomerase activity [1, 2]. Telomerase activity is only detectable in human stem cells and in most cancer cells which confer unlimited replication potential to these cells, therefore, telomerase has become an attractive target for antitumor therapy [3, 4].

In addition to hTERT and hTERC, multiple regulatory proteins are also found to associate with hTERT and/or hTERC which are essential for the telomerase holoenzyme assembly and activity [5, 6]. Several hTERT-associated proteins, such as EST1A [7], ATPase Pontin and Reptin [8], and molecular chaperones p23, and heat-shock protein 90 (Hsp90) [9] are required for the telomerase assembly and activity. The telomerase Cajal body protein 1 (TCAB1) associates with the 3′-end of hTERC which controls telomerase trafficking and telomere synthesis [10]. Several accessory proteins including DKC1 (dyskerin), NOP10, NHP2, and GAR1 bind to the 3′-end of hTERC H/ACA domain which are necessary for telomerase RNP biogenesis and localization [11–13]. Unlike the 3′-end region, the 5′-end region of hTERC contains several tandem G-tracts that are known to adopt G-quadruplex structure in vitro [14]. G-quadruplex formation in the 5′-end region of hTERC hinders telomerase template boundary-defining element P1 helix formation which results in misincorporation of inappropriate nucleotides into telomeres [15–17]. DHX36 (also known as RHAU), a member of the DEXH box family of RNA helicase which can bind and unwind both DNA and RNA G-quadruplexes, is reported to interact with the 5′-end G-rich region of hTERC to promote P1 helix template boundary formation and telomerase function by unwinding G-quadruplexes in hTERC [18–20]. However, whether other RNA binding proteins exist that can associate with the 5′-end region of hTERC to regulate telomerase function is still elusive.

The heterogeneous nuclear ribonucleoproteins F/H (hnRNP F/H) subfamily comprises hnRNP F, H1, H2, H3, and G-rich sequence factor 1 (GRSF1). The amino acid sequence of hnRNP H1 is 96 and 68% identical to hnRNP H2 and hnRNP F, respectively [21, 22]. HnRNP F/H possess three quasi-RNA recognition motif (qRRM) which preferentially bind to poly(G)-rich sequences RNA in the target exons and/or adjacent introns, thereby regulating alternative splicing and 3′-end processing of numerous genes [23–29]. However, whether hnRNP F/H can associate with the 5′-end region of hTERC and modulate telomerase function remains completely unknown.

In this study, we report that hnRNP F/H associate with both hTERC and telomerase holoenzyme to regulate telomerase activity and telomere length. Moreover, hnRNP F/H also contribute to cancer cell proliferation, migration, and invasion, as well as the proliferation and senescence of human mesenchymal stem cells (hMSCs).

## Results

### HnRNP F/H interact with the 5′-end region of hTERC in vitro and in vivo

**Fig. 1:**
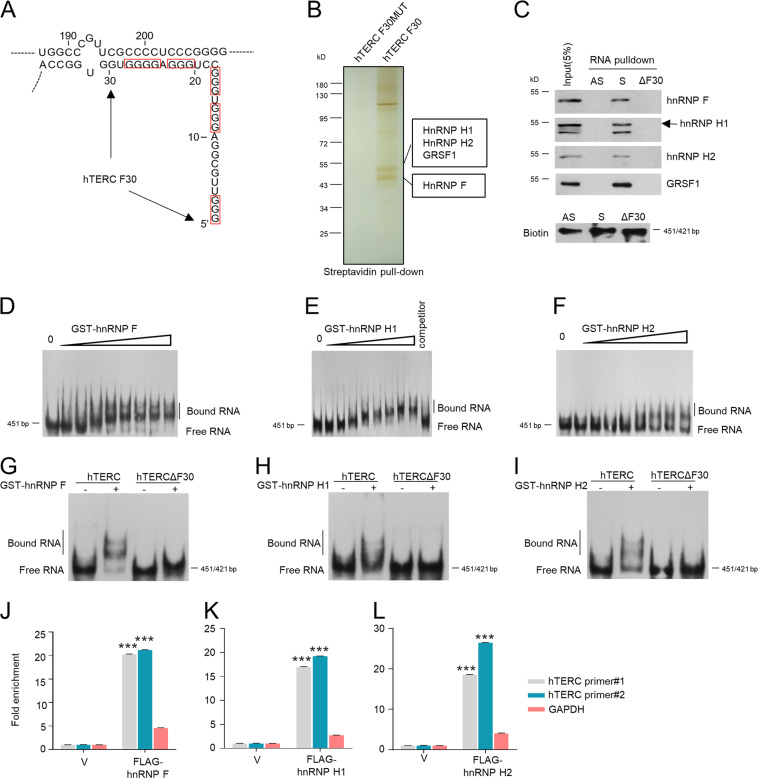
HnRNP F/H bind to 5′-region of hTERC in vitro and in vivo. **a** Schematic representation of 5′-region of hTERC containing five guanine runs marked by the red box. The truncation containing nucleotide residues from 1 to 30 (5′-3′) of hTERC was named as F30. **b** hnRNP F/H were pulled down by F30. HeLa cell extract incubated with biotin-labeled F30 or F30MUT and streptavidin-coupled beads. The elutes were separated by SDS-PAGE and silver-stained. The protein bands were retrieved and analyzed by mass spectrometry. The two boxes point to the hnRNP F/H signals. **c** Biotinylated full length of hTERC (sense, S), its antisense (AS), and hTERC mutant lacking F30 (ΔF30) were subjected to biotin pull-down assay to detect bound hnRNP F/H by Western blot. 5% of whole cell extracts used in reactions as input. The amount of biotinylated probes was detected by the Chemiluminescent Nucleic Acid Detection Module Kit (Thermo Fisher Scientific). **d**–**f** Biotinylated hTERC (1.5 nM) was incubated without protein (0 μg) or with increasing amounts (0.25, 0.5, 0.75, 1.0, 1.25, 1.5, 1.75, 2 μg) of GST-hnRNP F, H1, or H2, respectively. The reaction mixtures were separated on 4% native PAGE. 200-fold molar excess of unlabeled hTERC combined with 2 μg protein served as the competitor to prevent hnRNP F/H-hTERC interactions. **g**–**i** Biotinylated hTERC or hTERCΔF30 (1.5 nM) was incubated without protein (–) or with 2 μg of GST-hnRNP F, H1, or H2, respectively. The reaction mixtures were separated on 4% native PAGE. **j**–**l** hTERC is immunoprecipitated by FLAG-hnRNP F/H. Total cell extracts from HeLa cells stably expressed FLAG-hnRNP F, H1, H2, or empty vector were subjected to RNP-IP assays using FLAG antibody. Real-time qPCR analyzed the immunoprecipitated RNA. Two pairs of primers were used for hTERC. The enrichment for hTERC was normalized to the vector. GAPDH mRNA was used as a nonspecific binding control. Error bars represent means ± SD (*n* = 3). Statistical analysis was performed using Student′s *t* test (***P < 0.001).

The 5′-end region of hTERC contains several G-tracts that are known to form G-quadruplex in vitro [1] (Fig. 1a). To better understand the function of 5′-end region of hTERC and identify the novel hTERC-binding proteins, we first employed RNA-pulldown assay followed by mass spectrometry analysis using the biotinylated first 30 nucleotides of hTERC (hTERC F30) as a bait to pull down associated proteins. Mass spectrometry analysis indicated that four candidate proteins hnRNP F, H1, H2, and GRSF1, which all belong to the hnRNP F/H subfamily, were in the presence of the F30 interactome (Fig. 1b).

We then confirmed the interaction between hTERC and hnRNP F/H by using RNA-pulldown assay. Only biotinylated hTERC sense was able to interact with endogenous hnRNP F/H and GRSF1 instead of hTERC antisense (Fig. 1c). The sense mutant lacking of the 5′-end first 30 nucleotides (hTERC ∆F30) abolished the interaction, indicating that hTERC F30 is required for the association with hnRNP F/H. Similar results were obtained that only hTERC sense bound to the exogenous hnRNP F/H and GRSF1 (Supplementary Fig. 1A). Because of the mitochondrial localization of GRSF1, we decided to exclude GRSF1 in this study.

The direct binding between hTERC and hnRNP F/H was further confirmed by in vitro electrophoretic mobility shift assay (EMSA). The results showed that hnRNP F/H may form stable ribonucleoprotein complex with hTERC through direct binding (Fig. 1d–f; Supplementary Fig. 1B, C, D), whereas no association was detected between GST protein and hTERC or between hnRNP F/H proteins and hTERC antisense (Supplementary Fig. 1E–H). Furthermore, the binding of hnRNP F/H to hTERC ∆F30 was totally impaired (Fig. 1g–i). RNP-IP results further showed hTERC was dramatically enriched in hnRNP F/H-IP samples (Fig. 1j–l). Collectively, these results confirm the hTERC-hnRNP F/H interaction in vitro and in vivo.

To dissect whether the binding of hnRNP F/H to hTERC depends on the 5′-end hTERC G-quadruplex formation, we synthesized the first 43 nt of hTERC (hTERC F43) which can form G-quadruplex, and then conducted EMSA with hnRNP F in the presence of K^+^ or Li^+^ cations, respectively. K^+^ is known to promote G4-structure formation, whereas Li^+^ disrupts G-quadruplex [18–20]. The association between hnRNP F and F43 was much stronger in the Li^+^ than in the K^+^ (Supplementary Fig. 1I), suggesting that hnRNP F/H may prefer to bind to the hTERC without G-quadruplex. Since DHX36 prefer binding to the hTERC with G4-structure [19–21], we then further explored what impact hnRNP F/H may have on the binding of DHX36 to hTERC. RNA-pulldown results displayed that deletion of either hnRNP F or H1 notably enhanced DHX36-hTERC sense association (Supplementary Fig. 1J). RNP-IP results also demonstrated that knockdown of either hnRNP F or H1 significantly increased the HA-DHX36-enriched hTERC (Supplementary Fig. 1K). Altogether, these results suggest that hnRNP F/H deletion may increase the hTERC G4-structure formation in vivo thus leading to the binding augment between DHX36 and hTERC. Therefore, hnRNP F/H may bind to the 5′-end G-rich region of hTERC to prevent G4 formation.

### The G-tracts of hTERC and qRRM1 domain of hnRNP F/H are required for the hnRNP F/H-hTERC interaction

**Fig. 2:**
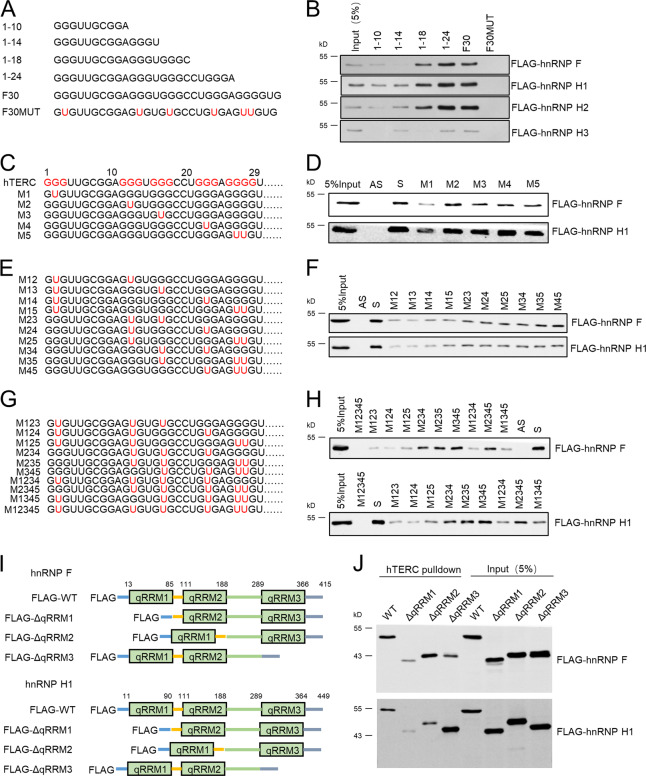
The guanine runs at 5′-end of hTERC and qRRM1 domain of hnRNP F/H mediate hnRNP F/H-hTERC interaction. **a** Nucleotide sequences of each F30 truncated RNAs used for the streptavidin pull-down assay. G to U substitutions made in F30MUT are marked by red. **b** 3′-biotinylated F30 truncations were incubated with HeLa (over-expressed FLAG-hnRNP F, H1, H2, or H3) whole cell extracts, respectively. The binding of hnRNP F/H to each RNA was assessed by western blotting. **c**, **e** and **g** Nucleotide sequences of each hTERC G to U mutants. G to U substitutions made in 5′-end of hTERC are marked by red. **d**, **f** and **h** Streptavidin pull-down assay was performed, and the binding of hnRNP F/H1 to each RNA was detected by western blot. Biotinylated antisense of hTERC served as a negative control. 5% of whole cell extracts used in reactions as input. **i** Schematic representation of three qRRM domains of hnRNP F/H1, as well as their truncations lacking of qRRM1, qRRM2, or qRRM3 domain, respectively. **j** Streptavidin pull-down assays were performed as aforementioned method, and the binding of hTERC to each hnRNP F/H1 truncations was detected by western blot. 5% of whole cell extracts used as input.

To clearly define the minimal sequence of hTERC required for hnRNP F/H binding, we generated synthetic RNAs with successive deletion of G-tracts from the 3′-end of hTERC F30 (Fig. 2a), and then performed RNA-pulldown assay. As shown in Fig. 2b, truncations 1–24 and F30 possessed the strong binding capability to hnRNP F/H, while the interaction of truncation 1–18 with hnRNP F/H reduced. Conversely, the truncations 1–14 and 1–10 almost completely lost the binding capacity to hnRNP F/H. In addition, G to U substitutions made in the five G-tracts of F30 (F30MUT) completely abolished the binding capacity to hnRNP F/H. These results indicate that the minimal sequence of hTERC capable of sufficient binding to hnRNP F/H may comprise the first three G-tracts of hTERC.

To further investigate the contribution of each G-tract in association with hnRNP F/H, we substituted the middle G to U in each of five G-tracts, respectively (named as M1–M5, Fig. 2c), or simultaneously substituted the middle G to U in two, three, four, or five G-tracts (named as M12, …, M123, …, M1234…, M12345, Fig. 2e, g). The RNA-pulldown and RNA-EMSA results showed that the binding ability of the M1 mutant to hnRNP F/H largely decreased compared with hTERC sense and the M2-M5 mutants (Fig. 2d, Supplementary Fig. 2A). We further observed that regardless of two, three, or four G to U substitutions, as long as the substitution containing the M1 mutation site, its binding ability to hnRNP F/H was then much weaker than the other corresponding substitutions. In contrast, the substitutions except containing the M1 mutation site, preserved substantial binding capability to hnRNP F/H (Fig. 2f, h). These results indicate that the first G-tract of hTERC may be the major motif that is needed to interact with hnRNP F/H. Substitution M12345 harboring all five G-tracts mutations completely lost the interaction with hnRNP F/H. Collectively, these data suggest that the G-tracts in the 5′-end region of hTERC are important for the association with hnRNP F/H, especially the first G-tract is bona fide required for the binding to the hnRNP F/H.

Each of hnRNP F, H1, and H2 contains three qRRM motifs (qRRM1–3) that are highly homologous in sequence (Fig. 2i; Supplementary Fig. 2B) [21–29]. To identify the hnRNP F/H domain requirements for hTERC interaction, we generated the individual qRRM motif truncated mutants of hnRNP F and H1 (Fig. 2i). RNA-pulldown results showed that the binding ability of ΔqRRM1 tuncations of hnRNP F and H1 to hTERC markedly decreased compared with the WT hnRNP F and H1 (Fig. 2j). HnRNP F ΔqRRM3 truncation and hnRNP H1 ΔqRRM2 truncation displayed mild decrease in association with hTERC. Interestingly, hnRNP H1 ΔqRRM3 truncation increased the interaction with hTERC. Altogether, these data imply that the qRRM1 domain is critical for hnRNP F/H1 binding to the hTERC.

### HnRNP F/H associate with telomerase holoenzyme complex

**Fig. 3:**
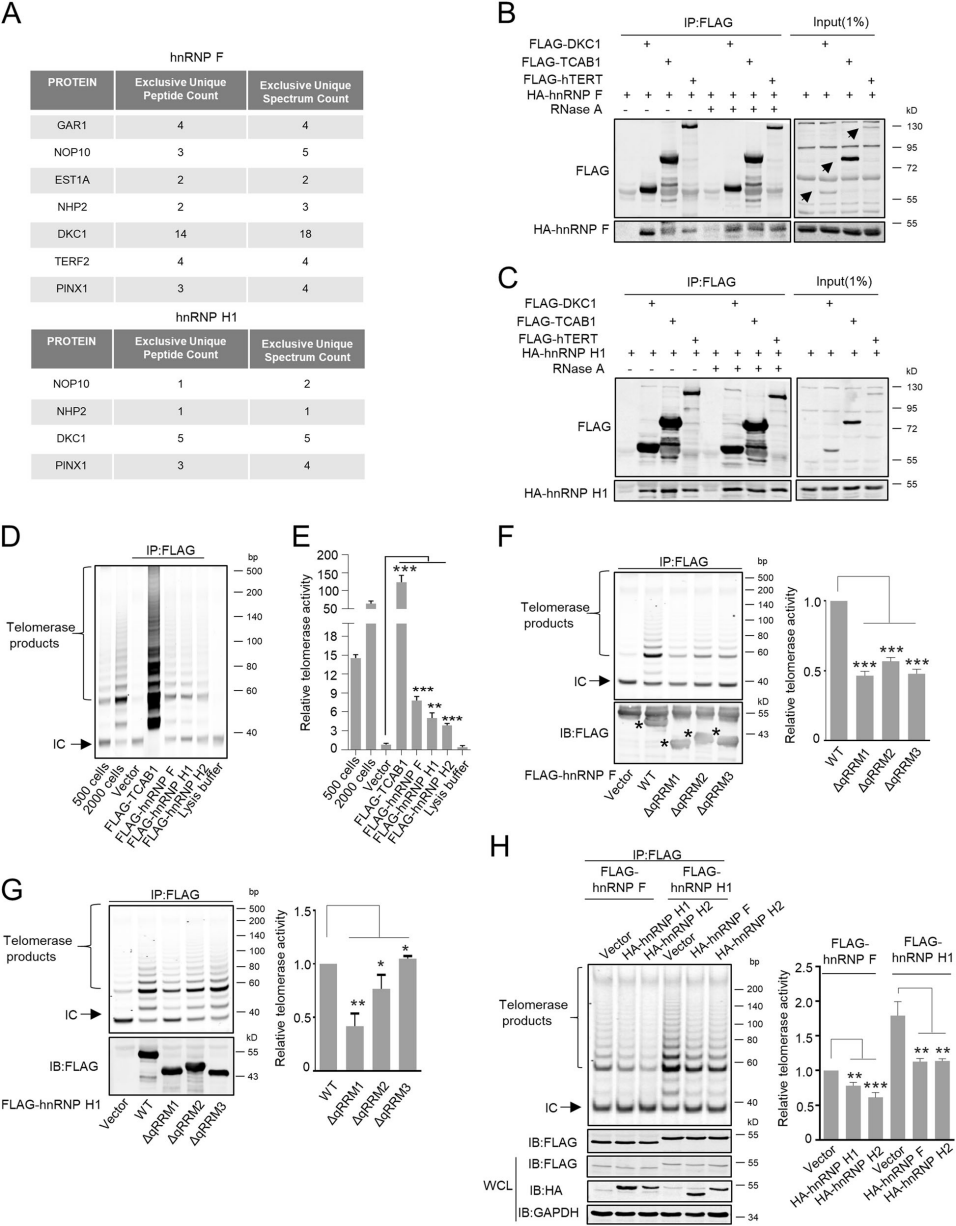
HnRNP F/H interact with telomerase holoenzyme complex. **a** Ectopically expressed FLAG-hnRNP F or H1 in HeLa cells were immunoprecipitated by FLAG antibody, and the immunoprecipitates were separated by SDS-PAGE and silver-stained. The protein bands were retrieved and analyzed by mass spectrometry. The numbers of unique peptide and unique spectrum of potential binding proteins implicated in telomerase holoenzyme are listed. HeLa cells transiently co-expressing HA-tagged hnRNP F (**b**) or H1 (**c**) with FLAG-tagged DKC1, TCAB1 or hTERT, respectively, were harvested and immunoprecipitated (IP) using anti-FLAG antibodies in the absence or in the presence of RNase A treatment. The immunoprecipitates were subjected to western blot analysis for the indicated proteins. Whole cell lysates served as input. **d** HeLa cell extracts stably overexpressed FLAG-tagged hnRNP F, H1, H2, or TCAB1 incubated with anti-FLAG antibodies and the immunoprecipitates (IP) were used for the TRAP assay to detect co-immunoprecipitated telomerase activity. The Vector served as a negative control, and FLAG-TCAB1 was used as a positive control. IC: Internal Control (36 bp). **e** Data from **d** were quantified and graphed. Error bars represent means ± SD (*n* = 3). Statistical analysis was performed using Student's *t* test (** *P* < 0.01, *** *P* < 0.001). HeLa cells transiently overexpressing FLAG tagged wild-type hnRNP F or its ΔqRRM trunctions **f,** H1 or its ΔqRRM trunctions **g**, were performed IP-TRAP to assess telomerase activity. The expressions of FLAG-hnRNP F/H1 and truncations were analyzed by Western blot. Vector served as a negative control. Error bars represent means ± SD (*n* = 3). Statistical analysis was performed using Student's *t* test (*P < 0.05, **P < 0.01, ***P < 0.001). **h** HeLa cells transiently co-expressing FLAG-tagged hnRNP F or H1 with HA-tagged indicated proteins were subjected to IP-TRAP. Error bars represent means ± SD (*n* = 3). Statistical analysis was performed using Student's *t* test (**P < 0.01, ***P < 0.001). WCL, whole cell lysate.

Many regulatory proteins are found to associate with hTERC and/or hTERT, such as Dyskerin complex, TCAB1, and EST1A, which are essential for the telomerase holoenzyme assembly and activity [5–13]. We sought to determine whether hnRNP F/H exclusively interact with hTERC or whether they also associate with the telomerase holoenzyme. In this regard, we performed Co-IP assay to immunoprecipitate FLAG-hnRNP F and H1 followed by mass spectrometry analysis (Supplementary Fig. 3A, B). Multiple telomerase holoenzyme components including GAR1, NOP10, DKC1, NHP2, EST1A, and shelterin complex component TERF2 were pulled down with hnRNP F (Fig. 3a). Similarly, NOP10, NHP2, and DKC1 were also immunoprecipitated by hnRNP H1.

Co-IP and GST-pulldown results confirmed that either hnRNP F or H1 associated with DKC1, TCAB1, and hTERT, respectively (Fig. 3b, c, Supplementary Fig. 3C). These associations were not disrupted when treated with RNaseA, indicating that the interactions may be independent of hTERC. Moreover, immunofluorescent results also revealed that hnRNP F colocalized with DKC1, TCAB1, and hTERT in the nuclei (Supplementary Fig. 3D).

As hnRNP F/H bound to the hTERC and directly associated with telomerase holoenzyme, we sought to test whether hnRNP F/H could recover telomerase activity using IP-TRAP assay. As shown in Fig. 3d, e, immunoprecipitated FLAG-tagged hnRNP F, H1, or H2 all constituted substantial telomerase activities from telomerase-positive HeLa cells, although their recovering telomerase activities were not as strong as the FLAG-TCAB1 immunopurified telomerase activity which served as a positive control [10].

Since qRRM1 deletion caused dramatic decrease of the binding between hnRNP F/H1 and hTERC, we further examined the impact of qRRM1 of hnRNP F/H1 on its recovering telomerase activity. Immunopurified FLAG-tagged ΔqRRM1 truncations retrieved significant less of telomerase activities (Fig. 3f, g), indicating that the binding impairment between them may have significant impact on the telomerase holoenzyme assembly and activity. In addition, the recovered telomerase activities by ΔqRRM2 and ΔqRRM3 truncations of hnRNP F as well as ΔqRRM2 truncation of hnRNP H1 also significantly reduced. Interestingly, the hnRNP H1 ΔqRRM3 truncation-recovered telomerase activity significantly elevated (Fig. 3g), which is consistent with the RNA-pulldown result (Fig. 2j). The explanation for these results needs to be further explored in the future.

Since hnRNP F, H1, and H2 all had ability to bind to the hTERC and telomerase complex, we then further explored what kind of relationship are they have in modulating telomerase activity. Overexpression of hnRNP H1 or H2 significantly reduced hnRNP F-retrieved telomerase activity. Similarly, hnRNP F or H2 overexpression also substantially attenuated hnRNP H1-recovered telomerase activity (Fig. 3h). These results suggest that hnRNP F/H may have competitive relationship to bind to the telomerase complex.

### HnRNP F/H knockdown impairs telomerase activity

**Fig. 4:**
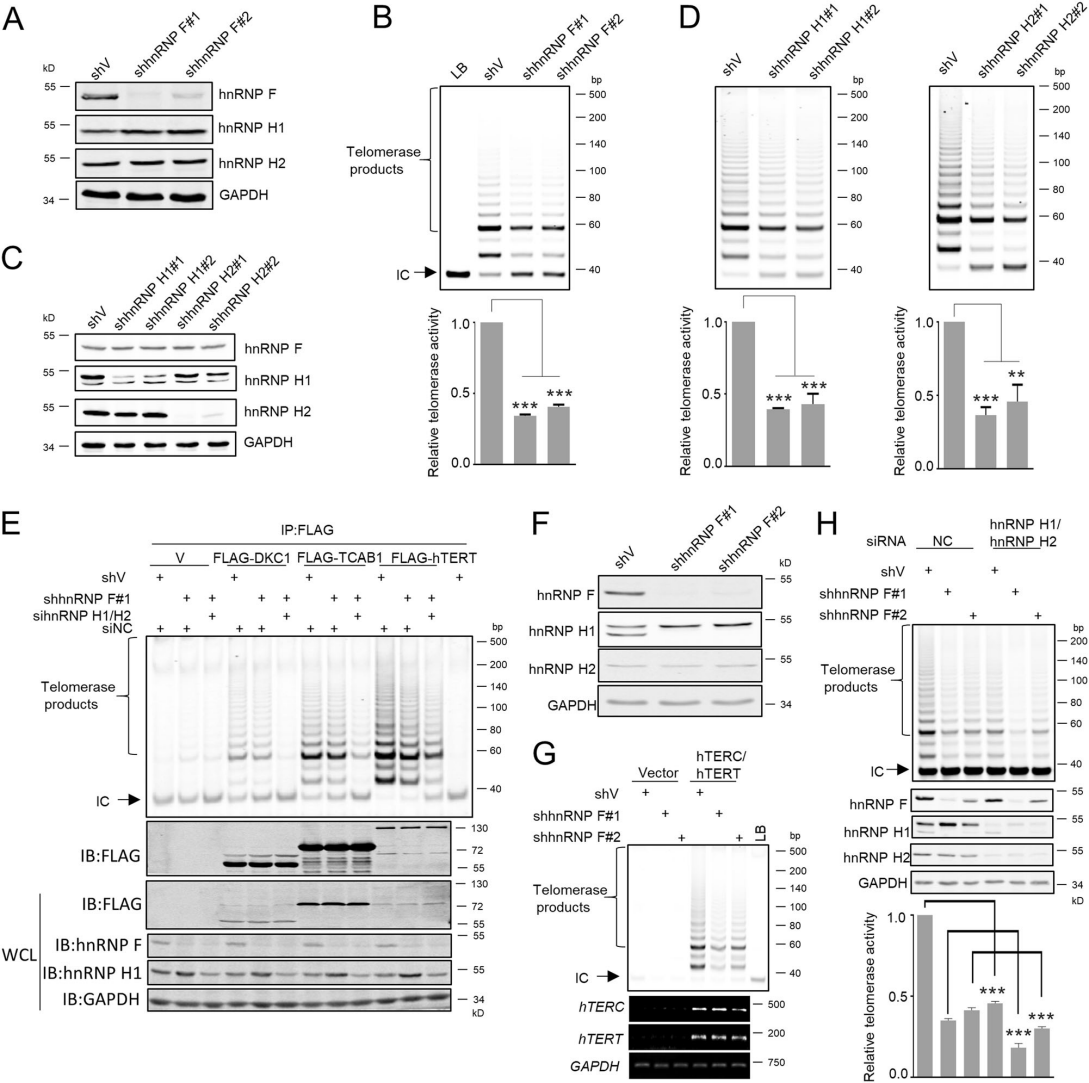
HnRNP F/H knockdown impairs telomerase activity. **a** HeLa cell lysates stably expressing control shRNA (shV) or hnRNP F shRNAs (#1 and #2) were examined for the indicated proteins by WB. **b** TRAP assays were performed in cell extracts described in **a**. 500 cells/μl were lysed and 2 μl was used for TRAP assay. IC: Internal Control (36 bp). LB: Lysis Buffer. Error bars represent means ± SD (*n* = 3). Statistical analysis was performed using Student′s *t* test (***P < 0.001). **c** HeLa cell lysates stably expressing control (shV), hnRNP H1 shRNA (#1 and #2), or hnRNP H2 shRNA (#1 and #2) were subjected to WB analysis. **d** TRAP assays were performed in cell extracts described in **c**. Error bars represent means ± SD (*n* = 3). Statistical analysis was performed using Student's *t* test (**P < 0.01, ***P < 0.001). **e** HeLa cells stably expressing pLKO.1-vector or pLKO.1-hnRNP F (#1 or #2) co-transfected with siNC or sihnRNP H1/H2 for 72 h, and then transfected with vector or FLAG-tagged DKC1, TCAB1, or hTERT plasmids for 48 h. Then cells were harvested for IP-TRAP assays and WB analysis. WCL, whole cell lysate. **f** U2OS cell extracts stably expressing control (shV) or hnRNP F shRNA (#1 and #2) were subjected to WB analysis. **g** U2OS cells described in **f** were transfected with vector or co-transfected with hTERC and hTERT. 500 cells/μl were lysed and 2 μl was used for TRAP assay. *hTERC*, *hTERT*, and *GAPDH* RNAs were detected by RT-PCR. **h** HeLa cells described in **a** transfected with NC (negative control) or hnRNP H1/hnRNP H2 siRNAs were harvested for TRAP assay and WB analysis. Error bars represent means ± SD (*n* = 3). Statistical analysis was performed using Student's *t* test (***P < 0.001).

To further exploit the role of hnRNP F/H in telomerase function, we generated hnRNP F, H1, and H2 stably knockdown (KD) HeLa cell lines, respectively (Fig. 4a, c). Deletion of hnRNP F, H1, or H2 resulted in an approximately half reduction in telomerase activity as measured by TRAP (Fig. 4b, d). Nevertheless, overexpression of hnRNP F, H1, or H2 did not further augment telomerase activities (Supplementary Fig. 4A), this may be due to the endogenous hnRNP F/H are already sufficient to support full telomerase activity in vivo.

It is well established that DKC1 binds to the 3′-end of hTERC H/ACA domain, and TCAB1 binds to the 4 nt CAB box within H/ACA domain [10–13]. Given that the association of hnRNP F/H with DKC1 and TCAB1 (Fig. 3a-c), we further examined the impact of hnRNP F/H on immunopurified FLAG-tagged DKC1, TCAB1, or hTERT-recovered telomerase activities. Stable depletion of hnRNP F partially attenuated DKC1, TCAB1, and hTERT-recovered telomerase activities, while hnRNP F, H1, and H2 triple knockdown markedly reduced telomerase activities (Fig. 4e). Nevertheless, individual overexpression of hnRNP F, H1, or H2 was unable to enhance DKC1, TCAB1, or hTERT-recovered telomerase activities (Supplementary Fig. 4B), which may be due to the endogenous hnRNP F/H are already sufficient to support full activity of telomerase.

We also coexpressed hTERC and hTERT to constitute recombinant telomerase in telomerase-negative U2OS cells. Similar to HeLa cell results, the recombinant telomerase activities in U2OS cells significantly reduced when hnRNP F or H1 was depleted (Fig. 4f, g; Supplementary Fig. 4C). Transiently knockdown of both hnRNP H1 and H2 in hnRNP F stable KD HeLa cells further decreased telomerase activity (Fig. 4h), indicating an additive effect of hnRNP F, H1, and H2 on the regulating telomerase activity. The rescue experiments showed that introduction of hnRNP F or H2 significantly restored telomerase activities in the hnRNP H1 KD cells (Supplementary Fig. 4D). Similar results were obtained by introducing hnRNP F or H1 in hnRNP H2 KD cells (Supplementary Fig. 4E). Collectively, these results imply that hnRNP F/H are important for the telomerase activity maintenance, and hnRNP F/H deficiency may cause a dramatic loss of telomerase activity.

### HnRNP F/H are critical for telomere length maintenance

**Fig. 5:**
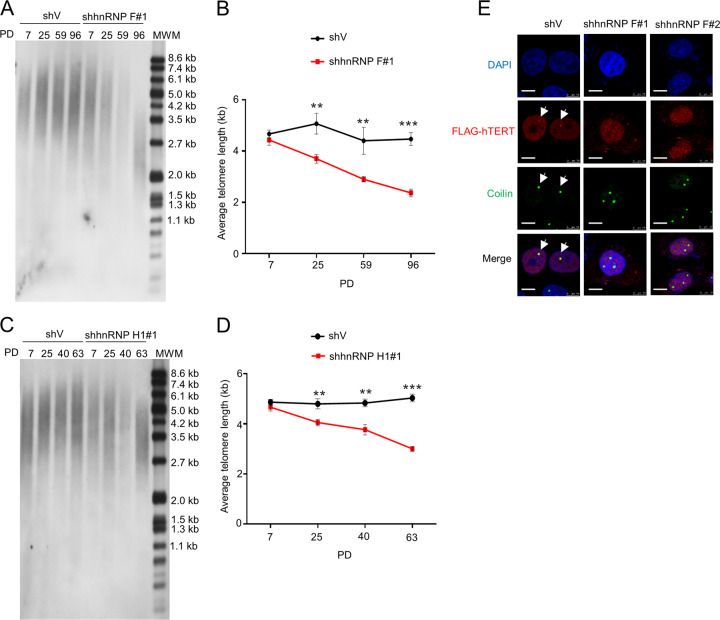
HnRNP F/H knockdown shortens telomere length and impairs telomerase assembly. **a** HeLa cells stably expressing control (shV) or hnRNP F shRNA (#1) were passaged over time and the average telomere length was examined at different population doublings (PD) by a telomere restriction fragment assay. **b** Data from **a** were quantified and graphed. Error bars represent means ± SD (*n* = 3). Statistical analysis was performed using Student's *t* test (**P < 0.01, ***P < 0.001). **c** HeLa cells stably expressing control (shV) or hnRNP H1 shRNA (#1) were passaged over time and the average telomere length was examined at different PD by a telomere restriction fragment assay. **d** Data from **c** were quantified and graphed. Error bars represent means ± SD (*n* = 3). Statistical analysis was performed using Student's *t* test (**P < 0.01, ***P < 0.001). **e** HeLa cells stably expressing control (shV) or hnRNP F shRNAs (#1 and #2) transfected with FLAG-hTERT plasmid for 48 h and then cells were stained for FLAG-hTERT using anti-FLAG antibody (red), Coilin (a specific Cajal body marker, green), and nuclei (DAPI, blue). Representative immunofluorescence images are shown. White arrow heads indicate the overlapped signals. Scale bar, 10 μm. The experiments were repeated three times.

We then inspected the role of hnRNP F/H in telomere length maintenance. We measured the average telomere length of serial passages of stable hnRNP F, H1, or H2 KD HeLa cells using telomere restriction fragment assay. The average telomere length in stable hnRNP F, H1, or H2 KD cells gradually shortened over time, whereas it remained stable at about 4.8 kb in control cells (Fig. 5a–d; Supplementary Fig. 5A–D). Especially at 96PD, the average telomere length in hnRNP F KD cells was shortened at least half compared with control cells (Fig. 5a, b). Collectively, these results suggest that hnRNP F/H play critical role in maintaining telomere length.

TCAB1 is critical for telomerase RNP distribution from nucleoli to Cajal bodies [10]. Given that hnRNP F/H interact with TCAB1 and hnRNP F/H deletion impairs TCAB1-recovered telomerase activity and leads to telomere shortening, we sought to probe whether hnRNP F/H have impact on the distribution of hTERT. The immunofluorescent signals of FLAG-hTERT overlapped with a specific Cajal bodies marker Coilin signals in the nuclei in both HeLa and U2OS control cells (Fig. 5e; Supplementary Fig. 5E). However, the overlap signals disappeared in both hnRNP F KD HeLa and U2OS cells. These results suggest that hnRNP F deficiency may disrupt the hTERT distribution to Cajal bodies thereby partly impairing the recruitment of telomerase to telomere and mitigating telomere synthesis.

### HnRNP F/H knockdown repress cancer cell proliferation, migration, and invasion

**Fig. 6:**
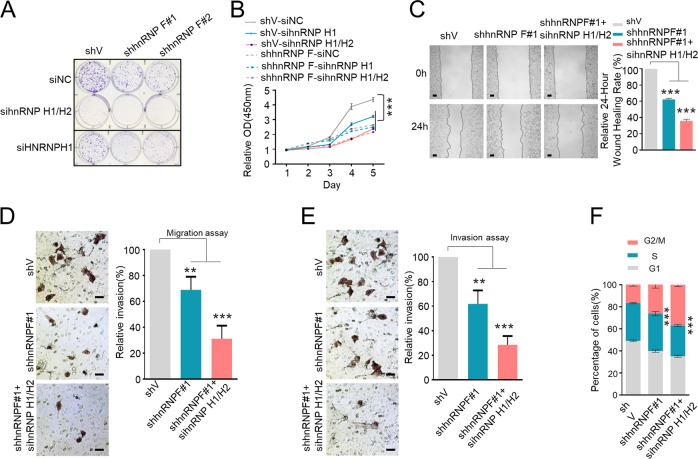
HnRNP F/H knockdown attenuate cancer cell proliferation, migration, and invasion. HeLa cells stably expressing pLKO.1-vector or pLKO.1-hnRNP F (#1 or #2) co-transfected with siNC, sihnRNP H1 or sihnRNP H1/H2, and then **a** colony formation assay was performed, **b** cell proliferation was determined by CCK-8 assay. **c** HeLa cells stably expressing pLKO.1-vector, or pLKO.1-hnRNP F (#1), or hnRNP F (#1) co-transfected with sihnRNP H1/H2, were subjected to wound healing assay. Scale bar, 0.1 mm. Data were quantified and graphed. Error bars represent means ± SD (*n* = 3). Statistical analysis was performed using Student's *t* test (***P < 0.001). HeLa cells as described in **c** were subjected to the transwell migration assay (**d**), transwell invasion assay (**e**), and cell cycle analysis (**f**). Scale bar, 10 μm. Error bars represent means ± SD (*n* = 3). Statistical analysis was performed using Student's *t* test (**P < 0.01, ***P < 0.001).

Approximately 85–90% of human cancer cells have reactivated telomerase activity to avoid cell senescence and ensure unlimited cell proliferation [30]. Considering the ability of hnRNP F/H to regulate telomerase activity and telomere length, we further inspected the effect of hnRNP F/H on cancer cell proliferation, migration, and invasion. HnRNP F KD and si-hnRNP H1 HeLa cells proliferation rate significantly reduced as measured by colony formation assay and CCK-8 assay (Fig. 6a, b; Supplementary Fig. 6A). More severe retardation of cell proliferation was observed after knockdown of hnRNP H1 in hnRNP F KD HeLa cells. Furthermore, knockdown of both hnRNP H1 and H2 in hnRNP F KD HeLa cells further reduced cell proliferation (Fig. 6a, b), indicating an additive effect of hnRNP F, H1, and H2 on regulating cancer cell proliferation. In addition, hnRNP F deletion also significantly suppressed cell motility, migration, and invasion (Fig. 6c–e). Similarly, triple knockdown of hnRNP F, H1, and H2 caused more profound decreases of cell motility, migration, and invasion. We also found that hnRNP F depletion caused significant G2/M accumulation and triple knockdown of hnRNP F, H1, and H2 triggered more obvious G2/M arrest (Fig. 6f). Altogether, these results suggest that hnRNP F/H exert great impact on cancer cell proliferation, migration, and invasion.

### HnRNP F deletion mitigates hMSC telomerase activity and cell proliferation, and accelerates cell senescence

**Fig. 7:**
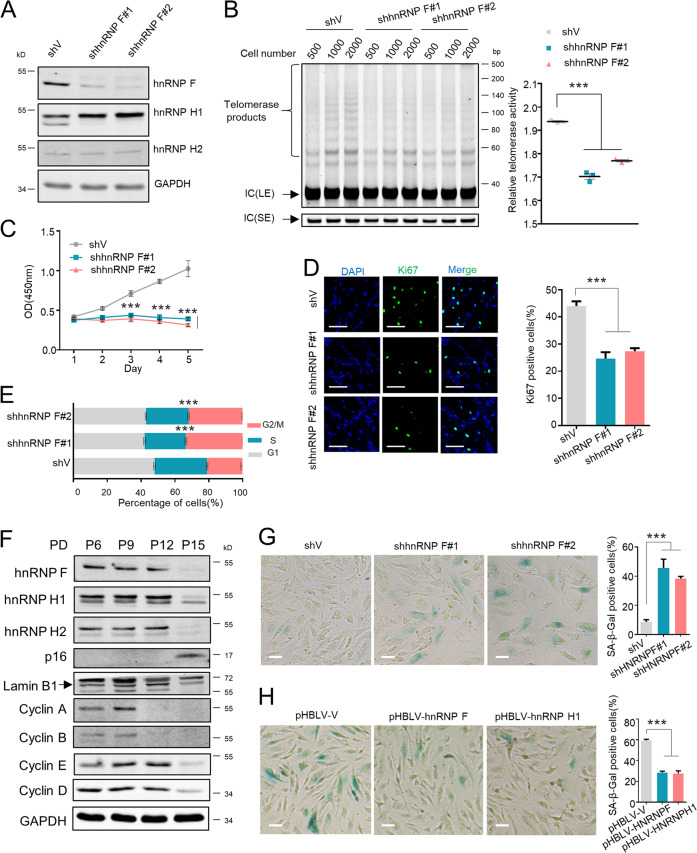
HnRNP F knockdown represses hMSCs telomerase activity and cell proliferation, and promotes cell senescence. **a** hMSCs cells stably expressing pLKO.1-vector or pLKO.1-hnRNP F (#1 or #2) were generated and hnRNP F knockdown was confirmed by WB analysis. Then the stable hMSCs cell lines were used to perform following assays: **b** TRAP assay, data were quantified and graphed. Error bars represent means ± SD (*n* = 3). Statistical analysis was performed using Student's *t* test (***P < 0.001); **c** CCK-8 cell proliferation assay, error bars represent means ± SD (*n* = 3). Statistical analysis was performed using Student's *t* test (***P < 0.001); **d** Cells were stained for Ki67 using anti-Ki67 antibody (green), and nuclei (DAPI, blue). Representative immunofluorescent images are shown. Scale bar, 100 μm. The experiments were repeated three times. Data were quantified and graphed. Error bars represent means ± SD (*n* = 3). Statistical analysis was performed using Student's *t* test (*** *P* < 0.001); **e** Cell cycle analysis, error bars represent means ± SD (*n* = 3). Statistical analysis was performed using Student's *t* test (***P < 0.001). **f** hMSCs cells passaged over time and cell lysates of different PDs were subjected to the WB analysis for the indicated proteins. **g** hMSCs cells described in **a** were assessed by SA-β-gal activity assay. Scale bar, 10 μm. Data were quantified and graphed. Error bars represent means ± SD (*n* = 3). Statistical analysis was performed using Student's *t* test (*** *P* < 0.001). **h** hMSCs cells stably expressing pHBLV-vector or pHBLV-hnRNP F or H1 were subjected to the SA-β-gal activity assay. Scale bar, 10 μm. Data were quantified and graphed. Error bars represent means ± SD (*n* = 3). Statistical analysis was performed using Student's *t* test (***P < 0.001).

hMSCs exhibit telomerase activity [31]. However, hMSCs have limited proliferative potential due to cells rapidly reaching replicative senescence as their telomeres progressively shorten with repeated cell divisions. hTERT overexpression restores telomerase activity and increases hMSCs life span [32, 33]. Taking this advantage, we further exploited the role of hnRNP F/H in hMSCs telomerase activity, proliferation, and senescence. For this end, hnRNP F stably knockdown cell lines were generated (Fig. 7a; Supplementary Fig. 7A). Similar to HeLa cells, hnRNP F deletion significantly reduced hMSCs telomerase activity (Fig. 7b), drastically inhibited hMSCs proliferation and Ki67-positive cell numbers (Fig. 7c, d), and caused significant hMSCs G2/M cell cycle arrest (Fig. 7e).

We also found that hnRNP F/H expression levels remained relatively stable before 12PDs, but almost lost when cells reaching senescent stage at 15PDs, which characterized by p16 upregulation and Lamin B1 loss (Fig. 7f). Accordingly, the expressions of cyclin A, B, D, and E all lost when hMSCs approaching to senescent state. Moreover, knockdown of hnRNP F in 5PDs of hMSCs largely accelerated cell senescence, which indicated by the dramatic increase of SA-β-gal activity (Fig. 7g). Conversely, hnRNP F or H1 overexpression in 12PDs of hMSCs remarkably retarded cell senescence (Fig. 7h). Meanwhile, hnRNP F or H1 overexpression also significantly elevated Ki67-positive cell numbers (Supplementary Fig. 7B), indicating that enforced expression of hnRNP F/H preserve hMSCs proliferation capacity. Collectively, these results suggest that hnRNP F/H modulate telomerase activity, cell proliferation, and senescence of hMSCs.

## Discussion

The 5′-end region of hTERC contains tandem G-tracts known to form G-quadruplex, which is resolved by the RNA helicase DHX36 [18–20]. Other than DHX36, no any other protein is identified to be able to interact with 5′-end of hTERC so far. Here, we found that hnRNP F/H directly associated with 5′-end G-rich region of hTERC (Figs. 1, 2; Supplementary Fig. 1, 2). Similar to DHX36, the hnRNP F/H-hTERC interaction required the first three G-tracts of hTERC and qRRM1 domain of hnRNP F/H. Importantly, the first G-tract of hTERC is bona fide required for the binding to hnRNP F/H (Fig. 2). But unlike to DHX36, hnRNP F/H might prefer to bind to the 5′-end of hTERC without G4 formation (Supplementary Fig. 1I). In addition, unlike to DHX36, hnRNP F/H also directly interacted with telomerase holoenzyme independent of hTERC (Fig. 3; Supplementary Fig. 3). Collectively, we identified hnRNP F/H as the novel accessory proteins bound to the 5′-end G-rich region of hTERC.

We demonstrated that immunopurified hnRNP F/H reconstituted a proportion of telomerase activity (Fig. 3d). Importantly, the individual knockdown of either hnRNP F or H largely attenuated telomerase activities, while the triple knockdown of hnRNP F/H further mitigated telomerase activities (Fig. 4; Supplementary Fig. 4). HnRNP F/H deletion also shortened telomere length over time (Fig. 5a–d; Supplementary Fig. 5A–D). All of these results suggest that hnRNP F/H may be the telomerase RNP components thus playing important roles in modulating telomerase activity and telomere length.

As for the underlying mechanism of how hnRNP F/H modulate telomerase activity and telomere length, one possible is that the hnRNP F/H-hTERC interaction may be important for preventing hTERC G4-RNA formation to ensure the P1 helix formation and the proper telomere synthesis since hnRNP F/H preferred to bind to the 5′-end of hTERC without G4 formation (Supplementary Fig. 1I) and hnRNP F/H deletion enhanced DHX36-hTERC interaction (Supplementary Fig. 1J, K). Hence, hnRNP F/H deficiency may promote G4-RNA formation thus abrogating P1 helix formation and interfering the proper telomere synthesis. Moreover, hnRNP F/H may be the telomerase RNP components thus contributing to the telomerase activity. Furthermore, hnRNP F knockdown disrupted the localization of hTERT to Cajal bodies (Fig. 5e; Supplementary Fig. 5E), which may impair the recruitment of telomerase to telomere thereby hindering telomere synthesis. All of these possibilities are needed to further investigate in the future.

Accumulating evidences have shown that hnRNP F/H are aberrantly expressed in a number of human cancers [34]. Alternative splicing of Bcl-2 family genes Mcl-1 and Bcl-x, a-raf gene, fructokinase (KHK) gene, RON gene, and EWS-FLI1 transcripts by hnRNP F/H are reported to contribute to various cancer cells proliferation, metastasis, and apoptosis-resistance [26, 35, 36]. HnRNP F/H-mediated alternative splicing of TCF3 also play important role in controlling stem cell pluripotency and differentiation [37]. We showed that hnRNP F/H knockdown strongly inhibited cancer cell proliferation, migration, and invasion, and greatly inhibited hMSCs proliferation and induced cell senescence (Figs. 6, 7; Supplementary Fig. 7). Therefore, other than acting as the splicing factor, our data provide the novel insights that hnRNP F/H control cancer cell growth and stem cell pluripotency via modulation of telomerase activity.

## Materials and methods

### Cell culture

HEK293T, HeLa, and U2OS cells were cultured in DMEM supplemented with 10% fetal bovine serum (FBS, Hyclone) and penicillin/streptomycin antibiotics. hMSCs were generously gifted from professor Guang-Hui Liu (Chinese Academy of Sciences), and were cultured in αMEM medium (Thermo Scientific, 32571036) plus GlutaMAX supplemented with 10% FBS (Gibco), penicillin/streptomycin (Thermo Scientific, 15070063), and 1 ng/ml bFGF (Joint Protein Central, JPC). Cells were under standard cell culture conditions (37 °C, 5% CO_2_, humidified atmosphere) and passaged every 2–3 days by trypsinization at a confluency of 80–90%. All cell cultures were routinely checked for mycoplasma contamination and confirmed negative.

### Viral infection and transfection

For lentiviral short hairpin RNA (shRNA) infection, HEK293T cells were seeded at 50–60% confluency and co-transfected with either pLKO.1-vector or target gene pLKO.1-shRNA with packaging plasmid (psPAX2) and envelope plasmid (pMD2.G) using Lipofectamine 2000 (Invitrogen). Medium was changed 6 h later. After 48 h, the viral particles were harvested and filtered through a 0.45 μm filter, and then used to infect parental cells for 12 h. The stably infected cells were then selected using 2 μg/ml puromycin for 4–6 days.

Two independent shRNA sequences against each target genes are listed as below:

shhnRNP F#1: 5′-CCGGGCAATTAAGAGCAGTTATAATCTCGAGATTATAACT

GCTCTTAATTGCTTTTTT -3′;

shhnRNP F#2: 5′-CCGGAGCGACCGAGAACGACATTTACTCGAGTAAATGTC GTTCTCGGTCGCTTTTTT-3′;

shhnRNP H1#1: 5′-CCGGAGCTGAAGTTAGAACTCATTACTCGAGTAATGAGT TCTAACTTCAGCTTTTTT-3′;

shhnRNP H1#2: 5′-CCGGTTGCCCTTTGCCACGTTAAATCTCGAGATTTAACGT GGCAAAGGGCAATTTTT-3′;

shhnRNP H2#1: 5′-CCGGGCTTACTGTAAAGTGGAAGTTCTCGAGAACTTCCA CTTTACAGTAAGCTTTTT-3′;

shhnRNP H2#2: 5′-CCGGCCATGAGAGTACATATTGAAA CTCGAGTTTCAATA TGTACTCTCATGGTTTTT-3′.

For transient transfection, plasmids were transfected using either PEI or Lipofectamine 2000 (Invitrogen) reagent following the manufacturer's instructions. For PEI transfection, the indicated plasmids were mixed with serum-free medium and PEI, and incubated for 20 min at room temperature, and then added to the cells cultured in DMEM with FBS. After 6–8 h transfection, the medium was replaced with fresh medium, and transfected cells were harvested 48 h later. siRNAs transfections were carried out using Lipofectamine RNAiMAX reagent (Invitrogen, 13778–150) according to the manufacturer's protocol for 72 h, and the final concentrations of siRNAs were 10 nM. The siRNA sequence against hnRNP H1/H2 was: 5′-GGTCCAAATAGTCCTGACA-3′; and control siRNA was: 5′-UUCUCCGAAC GUGUCACGU-3′.

### Antibodies

Antibodies raised against the following proteins were used at the indicated concentrations for western blotting (WB) or immunofluorescence (IF): rabbit anti-hnRNP F antibody (Abcam, ab50982; 1:1000 for WB; 1:500 for IF); rabbit anti-hnRNP H1 antibody (Abcam, ab154894; 1:1,000 for WB); rabbit anti-hnRNP H2 antibody (Abcam, ab179439; 1:1000 for WB); rabbit anti-hnRNP H3 antibody (Abcam, ab66663; 1:1000 for WB); rabbit anti-GRSF1 antibody (Abcam, ab205531; 1:1000 for WB); rabbit anti-TCAB1 antibody (Novus, NB100–68252; 1:1000 for IF); mouse anti-coilin antibody (Abcam, ab87913; 1:1000 for IF); mouse anti-FLAG M2 antibody (Sigma, F3165; 1:10,000 for WB;1:1000 for IF); rabbit anti-FLAG antibody (Sigma, F7425; 1:10,000 for WB;1:1000 for IF); rabbit anti-HA-Tag antibody (Cell Signaling, 3724S; 1:3000 for WB); mouse anti-GST antibody (Abgent, AM1011b; 1:3000 for WB); rabbit anti-GAPDH antibody (Bioworld, AP0063; 1:10,000 for WB); mouse anti-Cyclin A2 antibody (Cell Signaling, 4656; 1:1000 for WB); rabbit anti-Cyclin B1 antibody (Cell Signaling, 4138; 1:1000 for WB); rabbit anti-Cyclin D1 antibody (Cell Signaling, 2978; 1:1000 for WB); mouse anti-Cyclin E1 antibody (Cell Signaling, 4129; 1:1000 for WB); mouse anti-Ki-67 antibody (Origene, ZM-0166). Secondary-antibody conjugates for IF included as following (concentrations in parentheses): goat anti-mouse Alexa Fluor 488 (Abcam, ab150117, 1:1000); goat anti-rabbit Alexa Fluor 488 (Abcam, ab150077, 1:1,000); goat anti-mouse Alexa Fluor 647 (Abcam, ab150115, 1:1000); goat anti-rabbit Alexa Fluor 594 (Abcam, ab150080, 1:1000).

### Confocal microscopy of fixed and live cells

Cells grown on glass coverslips were fixed by 4% paraformaldehyde for 10 min before permeabilized with 0.5% Triton X-100 for 10 min at room temperature. Coverslips were washed three times with PBS, and blocked with 5% BSA for 1 h at room temperature, and then incubated with the indicated antibodies overnight at 4 °C. The samples were washed three times with PBS and incubated with indicated secondary antibody (Abcam) for 1 h at room temperature. The samples were washed again with PBS and were stained with DAPI (Sigma, D9542). Immunofluorescence images were captured with a confocal laser scanning microscope.

### Western blotting

Cell pellets were lysed in RIPA buffer (Applygen Technologies) containing phosphatase inhibitor (Roche Diagnostics) and protease inhibitor cocktail (Amresco). Protein concentration was measured using BCA Protein Assay Kit (Thermo Scientific). Cell lysates (20–40 μg) were subjected to SDS-PAGE and transferred to nitrocellulose membranes (Millipore). The membrane was blocked using 5% milk in TBST buffer at room temperature for 1 h. Primary antibodies were blotted using 5% milk or BSA in TBST, and incubated at 4 °C overnight. Secondary antibodies (Dylight 800, Goat Anti-Rabbit IgG (H + L), EarthOx, E032820, or Goat Anti-Mouse IgG (H + L) EarthOx, E032810) were incubated for 1 h at room temperature in 5% milk/TBST. Then the signals were captured by Odyssey system.

### RNA isolation and real-time qPCR

Total RNA was isolated using the RNAiso Plus from Takara (9108) according to the manufacturer's description. 2 μg total RNA was transcribed into cDNA with the RevertAid First Strand cDNA Synthesis Kit (K1622, Fermentas) and subsequently analyzed using specific primers for hnRNP F, hnRNP H1, and hnRNP H2 mRNA and GAPDH primer as endogenous controls. Quantitative Real-Time PCR was carried out with an ABI 7500 Real-Time PCR system (Thermo Fisher Scientific). Relative RNA expression levels were calculated by the 2^−ΔΔCt^ method. The following primer pairs were used for Real-Time-PCR:

hnRNP F-F: 5′-GAGCTTCGGTGGAATTTCG-3′;

hnRNP F-R: 5′-GTAATGAAATGTCCACGGAGG-3′;

hnRNP H1-F: 5′-CTTGAATTCTACAGCAGGAGC-3′;

hnRNP H1-R: 5′-CTGGACTGGTTTGACAAGC-3′;

hnRNP H2-F: 5′-GGTATCGTTAGAGCTACACCA-3′;

hnRNP H2-R: 5′-CATCACTTCATCGGCTGAG-3′.

### TRAP, IP-TRAP, and telomere length measurement

Telomere repeat amplification protocol (TRAP) was used for determining telomerase activity [38]. Briefly, 50,000 HeLa or U2OS cells were harvested and lysed in ice-cold NP-40 lysis buffer at a concentration of 500 cells/μl. 2 μl of samples were added to reaction buffer and incubated at 30 °C for the extension of the substrate by telomerase. PCR was then performed to amplify the extension products. Following PCR, 5 μl of loading dye was add to each TRAP reaction mixture and 15 μl mixture was taken out and run on the 10% nondenaturing acrylamide gel in 0.5 × TBE (1.5 h, 110 V). Fixed the gel and visualized signals using Odyssey system. For IP-TRAP, 6 cm-dish transfected cells were harvested and lysed in ice-cold NP-40 lysis buffer. After centrifugation, the supernatant was incubated with anti-FLAG antibodies and the immunoprecipitates (IP) were used in TRAP assays to detect co-precipitated telomerase activities. For telomere length measurement, we used a kit from Roche (Cat. No. 12 209 136 001) and performed southern blot analysis following the manufacturer's instructions.

### Biotinylated RNA pull down assay and RNA-IP assay

Biotinylated RNA pull down assay and RNA-IP were performed as previously described [39]. To prepare templates for in vitro transcription, the following primer pairs were used: forward primers:

5′-(T7) GGGTTGCGGAGGGTGGGCCTGGGAGGGGT-3′ for sense hTERC;

5′-(T7) GCATGTGTGAGCCGAGTCC-3′ for antisense hTERC;

5′-(T7) GTGTTGCGGAGGGTGGGCCTGGGAGGGGT-3′ for M1;

5′-(T7) GGGTTGCGGAGTGTGGGCCTGGGAGGGGT-3′ for M2;

5′-(T7) GGGTTGCGGAGGGTGTGCCTGGGAGGGGT-3′ for M3;

5′-(T7) GGGTTGCGGAGGGTGGGCCTGTGAGGGGT-3′ for M4;

5′-(T7) GGGTTGCGGAGGGTGGGCCTGGGAGTTGT-3′ for M5;

5′-(T7) GTGTTGCGGAGTGTGGGCCTGGGAGGGGT-3′ for M12;

5′-(T7) GTGTTGCGGAGGGTGTGCCTGGGAGGGGT-3′ for M13;

5′-(T7) GTGTTGCGGAGGGTGGGCCTGTGAGGGGT-3′ for M14;

5′-(T7) GTGTTGCGGAGGGTGGGCCTGGGAGTTGT-3′ for M15;

5′-(T7) GGGTTGCGGAGTGTGTGCCTGGGAGGGGT-3′ for M23;

5′-(T7) GGGTTGCGGAGTGTGGGCCTGTGAGGGGT-3′ for M24;

5′-(T7) GGGTTGCGGAGTGTGGGCCTGGGAGTTGT-3′ for M25;

5′-(T7) GGGTTGCGGAGGGTGTGCCTGTGAGGGGT-3′ for M34;

5′-(T7) GGGTTGCGGAGGGTGTGCCTGGGAGTTGT-3′ for M35;

5′-(T7) GGGTTGCGGAGGGTGGGCCTGTGAGTTGT-3′ for M45;

5′-(T7) GTGTTGCGGAGTGTGTGCCTGGGAGGGGT-3′ for M123;

5′-(T7) GTGTTGCGGAGTGTGGGCCTGTGAGGGGT-3′ for M124;

5′-(T7) GTGTTGCGGAGTGTGGGCCTGGGAGTTGT-3′ for M125;

5′-(T7) GGGTTGCGGAGTGTGTGCCTGTGAGGGGT-3′ for M234;

5′-(T7) GGGTTGCGGAGTGTGTGCCTGGGAGTTGT-3′ for M235;

5′-(T7) GGGTTGCGGAGGGTGTGCCTGTGAGTTGT-3′ for M345;

5′-(T7) GTGTTGCGGAGTGTGTGCCTGGGAGGGGT-3′ for M1234;

5′-(T7) GGGTTGCGGAGTGTGTGCCTGTGAGTTGT-3′ for M2345;

5′-(T7) GTGTTGCGGAGGGTGTGCCTGTGAGTTGT-3′ for M1345;

5′-(T7) GTGTTGCGGAGTGTGTGCCTGTGAGTTGT-3′ for M12345;

5′-(T7) GTGGCCATTTTTTGTCTAACCCTAAC-3′ for ΔF30;

reverse primers: 5′-GCATGTGTGAGCCGAGTCC-3′ for hTERC or variants.

HnRNP H1: forward primer: 5′-(T7) ATGATGTTGGGCACGGAAGGTG-3′; and reverse primer: 5′-CTATGCAATGTTTGATTGAAAATCACTGG-3′. Short biotinylated RNAs were synthesized from GenePharma Company. The pulldown materials were subsequently analyzed by Western blotting or mass spectrometry analysis (MS). For identification of the proteins that interact with the 5′-end region of hTERC, we used synthesized biotinylated hTERC F30 and mutant negative control F30MUT RNA probes (400 ng) to incubate with HeLa cell lysate (1 mg), respectively, for RNA pull down assay. The pulldown materials were then separated by SDS‐PAGE and silver‐stained. The specific protein bands were recovered and analyzed by MS.

The following primer pairs were used for detecting hTERC levels in RNA-IP assays:

hTERC#1-F: 5′-CCCTAACTGAGAAGGGCGTA-3′;

hTERC#1-R: 5′-AGAATGAACGGTGGAAGGCG-3′;

hTERC#2-F: 5′-TCCACCGTTCATTCTAGAGCA-3′;

hTERC#2-R: 5′-GCTGACAGAGCCCAACTCTTC-3′.

### RNA EMSA

LightShift Chemiluminescent REMSA Kit (Pierce, 20158) was used to carry out RNA EMSA assays according to the manufacturer's protocol. RNA probes of TERC and variants were in vitro transcribed. Short biotinylated RNAs were synthesized from GenePharma Company. GST-fusion proteins were purified from prokaryotic system. The amount of probes or proteins used was indicated in figure legends.

### Other common assays

Cell proliferation assay, colony formation assay, wound healing assay, transwell migration and invasion assay, cell cycle analysis, and SA-β-gal activity assay were performed as previously described [40].

### Statistical analysis

In all experiments, data were presented as the mean ± SD. Unless otherwise stated, Student's *t* test was used to analyze statistical differences between groups. Statistical analyses were carried out using GraphPad (version 6.01). A two-tailed *P* value of <0.05 was considered significant. *P < 0.05, **P < 0.01, ***P < 0.001.

## Supplementary information

Supplemental Figure Legends

Supplemental Figure 1

Supplemental Figure 2

Supplemental Figure 3

Supplemental Figure 4

Supplemental Figure 5

Supplemental Figure 6

Supplemental Figure 7
