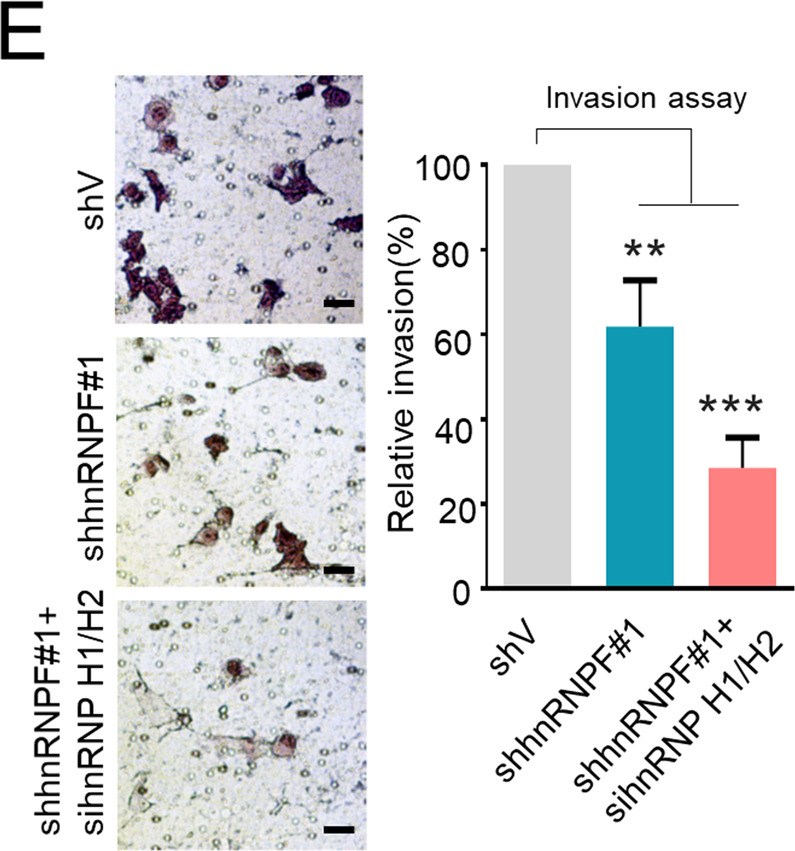# Correction to: HnRNP F/H associate with hTERC and telomerase holoenzyme to modulate telomerase function and promote cell proliferation

**DOI:** 10.1038/s41418-021-00806-y

**Published:** 2021-05-27

**Authors:** Chenzhong Xu, Nan Xie, Yuanyuan Su, Zhaomeng Sun, Yao Liang, Na Zhang, Doudou Liu, Shuqin Jia, Xiaofang Xing, Limin Han, Guodong Li, Tanjun Tong, Jun Chen

**Affiliations:** 1 Peking University Research Center on Aging, Beijing Key Laboratory of Protein Posttranslational Modifications and Cell Function, Department of Biochemistry and Molecular Biology, Department of Integration of Chinese and Western Medicine, School of Basic Medical Science, Peking University, 100191 Beijing, China; 2 Department of Physiology and Pathophysiology, School of Basic Medical Science, Peking University, 100191 Beijing, China; 3 Department of Molecular Diagnostics, Key Laboratory of Carcinogenesis and Translational Research (Ministry of Education), Peking University Cancer Hospital & Institute, 100142 Beijing, China

**Keywords:** RNA-binding proteins, RNA

Correction to: *Cell Death & Differentiation*

10.1038/s41418-019-0483-6.

The original version of this article unfortunately contained a mistake. Following publication of this article, the authors noticed that there was an error in the image used to compile the Fig. 6E shV image. An adjacent serial image section from Fig. 6D shV image was inadvertently used for Fig. 6E shV image during image compilation. The corrected images are provided below. The authors apologise for this error.