# Supplementary material for: HnRNP F/H associate with hTERC and telomerase holoenzyme to modulate telomerase function and promote cell proliferation
Source: Cell Death Differ. 2019 Dec 20;27(6):1998–2013. doi: 10.1038/s41418-019-0483-6 (PMC7244589; doi:10.1038/s41418-019-0483-6)
Supplement: Supplementary file 1 — Supplemental Figure Legends [file 41418_2019_483_MOESM1_ESM.docx]

**Supplementary Info to Xu et al. “HnRNP F/H associate with hTERC and telomerase holoenzyme to modulate telomerase function and promote cell proliferation”**

**Supplemental Figure 1.** HnRNP F/H bind to 5’-region of hTERC in vitro and in vivo.

(**A**) HeLa cells transfected with indicated plasmids were harvested and performed RNA pulldown using the biotinylated full length of hTERC (sense, S), its antisense (AS), and hTERC mutant lacking F30 (ΔF30) and detected bound FLAG-hnRNP F/H by Western blot. (**B**) The purified recombinant GST-tagged hnRNP F, H1 and H2 proteins were separated on SDS-PAGE gel and stained with coomassie blue. (**C**-**E**) Biotinylated hTERC (1.5 nM) was incubated without protein (0 μg) or with increasing amounts (2, 3, 4, 5, 6, 7, 8 μg) of GST-hnRNP F or H1, or with increasing amounts (0.5, 0.75, 1.0, 1.25, 1.5, 1.75, 2 μg) of GST proteins, respectively, for 30 min at room temperature and separated on 4% native PAGE. (**F**-**H**) Biotinylated hTERC antisense (1.5 nM) was incubated without protein (0 μg) or with increasing amounts (0.25, 0.5, 0.75, 1.0, 1.25, 1.5, 1.75, 2 μg) of GST–hnRNP F, H1 or H2, respectively, for 30 min at room temperature and separated on 4% native PAGE. (**I**) Biotinylated 43 nt fragment of 5’-end hTERC was incubated without protein (-) or with 1, 2 μg GST–hnRNP F proteins under standard 100 mM KCl (K^+^) or 100 mM LiCl (Li^+^) RNA EMSA conditions, then separated on 4% native PAGE. (**J**) HnRNP F and H1 stably knockdown (KD) HeLa cell lines were transiently overexpressed with HA-DHX36, respectively. Then the whole cell lysates were extracted and incubated with biotinylated hTERC (sense, S) and antisense (AS) to perform biotin pull-down assay. (**K**) Total cell extracts from HeLa cells described in (J) were subjected to RNP-IP assays using HA antibody. Real-time qPCR analyzed the immunoprecipitated hTERC. The enrichment for hTERC was normalized to the vector. GAPDH mRNA was used as a nonspecific binding control. Error bars represent means ± SD (n = 3). Statistical analysis was performed using Student’s t-test (** *P* < 0.01, *** *P* < 0.001).

**Supplemental Figure 2.** The guanine runs at 5’-end of hTERC are critical for the hnRNP F/H-hTERC interaction.

(**A**) Biotinylated G to U mutants (1.5 nM) made in the 5’-end of hTERC were incubated without protein (-) or with 2 μg GST-hnRNP F, H1, or H2 proteins under standard RNA EMSA conditions, then separated on 4% native PAGE. Biotinylated hTERC sense (1.5 nM) served as loading control. (**B**) Schematic representation of genome location, cellular location and sequence identity of hnRNP F/H family members.

**Supplemental Figure 3.** HnRNP F/H interact with telomerase holoenzyme complex.

(**A** and **B**) Ectopically expressed FLAG-hnRNP F or H1 in HeLa cells were immunoprecipitated by FLAG antibody, and the immunoprecipitates were separated by SDS-PAGE and silver-stained. The silver-stained images are shown. (**C**) Purified FLAG-tagged DKC1, TCAB1, or hTERT proteins from HeLa cells were incubated with 1 μg GST, GST-hnRNP F, H1, or H2 proteins to perform GST-pulldown assays and separated on SDS-PAGE. The indicated proteins were blotted. (**D**) HeLa cells transfected with FLAG-DKC1, TCAB1, or hTERT plasmids for 48 h and then cells were stained for FLAG- DKC1, TCAB1, or hTERT using anti-FLAG antibody (green), endogenous hnRNP F (red), and nuclei (DAPI, blue). Representative immunofluorescent images are shown. Scale bar, 10 μm. The experiments were repeated three times.

**Supplemental Figure 4.** HnRNP F/H modulate telomerase activity.

(**A**) HeLa cell lysates stably expressing control pHBLV (V) or pHBLV-FLAG-hnRNP F, H1, or H2 were subjected to IP-TRAP assays. Data were quantified and graphed. Error bars represent means ± SD (n = 3). Statistical analysis was performed using Student’s t-test. ns: no significance. (**B**) HeLa cells described in (A) co-transfected with FLAG-DKC1, TCAB1, or hTERT, respectively, then cell lysates were subjected to the IP-TRAP assays and WB analysis. WCL, whole cell lysate. (**C**) U2OS cell lysates stably expressing control (shV), hnRNP H1 shRNA (#1 and #2) were subjected to WB analysis and TRAP assays. Error bars represent means ± SD (n = 3). Statistical analysis was performed using Student’s t-test (*** *P* < 0.001). (**D** and **E**) HeLa cells stably expressing control (shV), hnRNP H1 shRNA (#1 and #2) (D), or hnRNP H2 shRNA (#1 and #2) (E), were transfected with FLAG-vector, hnRNP F, or H2, or H1, and harvested for TRAP assay and WB analysis. Error bars represent means ± SD (n = 3). Statistical analysis was performed using Student’s t-test (** *P* < 0.01, *** *P* < 0.001).

**Supplemental Figure 5.** HnRNP F/H regulate telomere length and telomerase assembly.

(**A**) HeLa cells stably expressing control (shV) or hnRNP F shRNA (#2) were passaged over time and the average telomere length was examined at different population doublings (PD) by a telomere restriction fragment assay. (**B**) Data from (A) were quantified and graphed. Error bars represent means ± SD (n = 3). Statistical analysis was performed using Student’s t-test (*** *P* < 0.001). (**C**) HeLa cells stably expressing control (shV) or hnRNP H2 shRNA (#1) were passaged over time and the average telomere length was examined at different PD by a telomere restriction fragment assay. (**D**) Data from (C) were quantified and graphed. Error bars represent means ± SD (n = 3). Statistical analysis was performed using Student’s t-test (* *P* < 0.05, *** *P* < 0.001). (**E**) U2OS cells stably expressing control (shV) or hnRNP F shRNAs (#1 and #2) transfected with FLAG-hTERT plasmid for 48 h and then cells were stained for FLAG-hTERT using anti-FLAG antibody (green), coilin (a specific Cajal body marker, red), and nuclei (DAPI, blue). Representative immunofluorescent images are shown. White arrows head indicate the overlapped signals. Scale bar, 10 μm. The experiments were repeated three times.

**Supplemental Figure 6.** HnRNP F/H knockdown slows down cancer cell cycle.

(**A**) HeLa cells stably expressing pLKO.1-vector or pLKO.1-hnRNP F (#1 or #2) co-transfected with siNC, sihnRNP H1 or sihnRNP H1/H2, then the indicated proteins were detected by WB.

**Supplemental Figure 7.** HnRNP F/H1 overexpression preserve hMSC cell proliferation ability. (**A**) Total RNAs were extracted from hMSC cells stably expressing control (shV) and hnRNP F shRNA (#1 and #2), and then subjected to RT-qPCR analysis for hnRNP F/H mRNA levels. Error bars represent means ± SD (n = 3). Statistical analysis was performed using Student’s t-test (*** *P* < 0.001). ns: no significance ( **B**) hMSC cells stably transfected with pHBLV-V, hnRNP F or H1, then cells were stained for Ki67 using anti-Ki67 antibody (green), and nuclei (DAPI, blue). Representative immunofluorescent images are shown. Scale bar, 10 μm. The experiments were repeated three times. Data were quantified and graphed. Error bars represent means ± SD (n = 3). Statistical analysis was performed using Student’s t-test (* *P* < 0.05; *** *P* < 0.001).
