# Supplementary figures and images for: HnRNP F/H associate with hTERC and telomerase holoenzyme to modulate telomerase function and promote cell proliferation
Source: Cell Death Differ. 2019 Dec 20;27(6):1998–2013. doi: 10.1038/s41418-019-0483-6 (PMC7244589; doi:10.1038/s41418-019-0483-6)

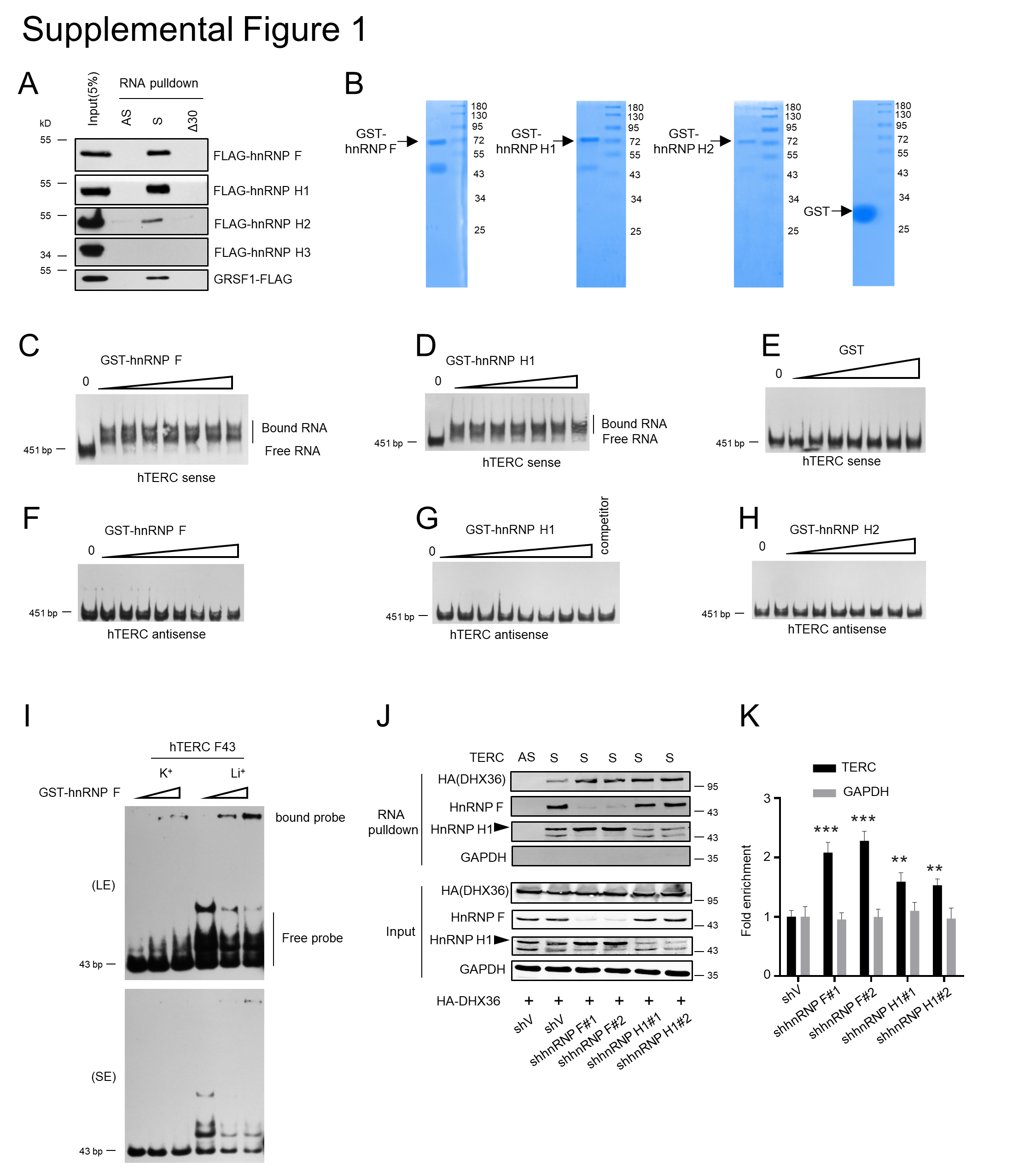

Supplement: Supplementary file 2 — Supplemental Figure 1 [file 41418_2019_483_MOESM2_ESM.tif]

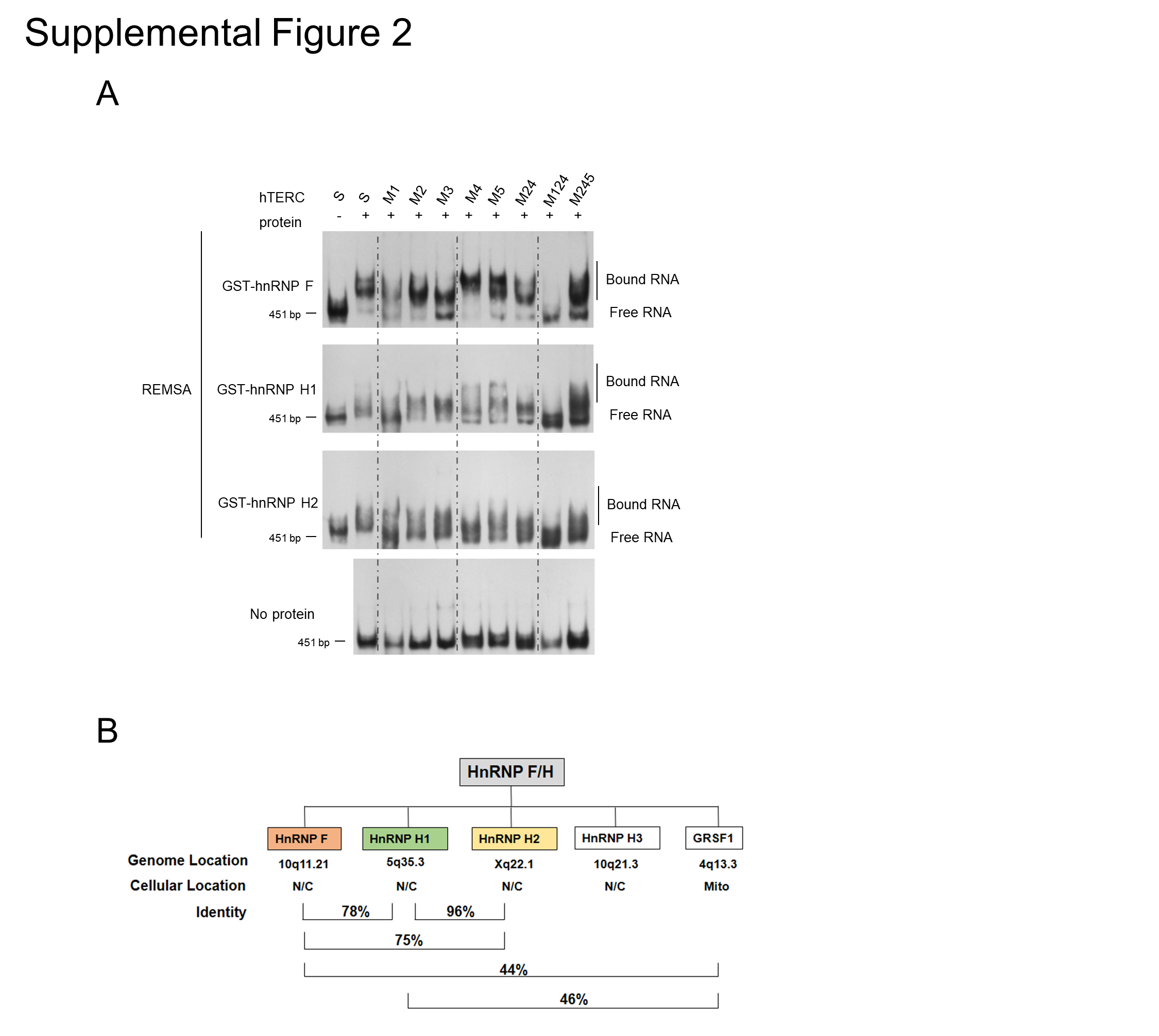

Supplement: Supplementary file 3 — Supplemental Figure 2 [file 41418_2019_483_MOESM3_ESM.tif]

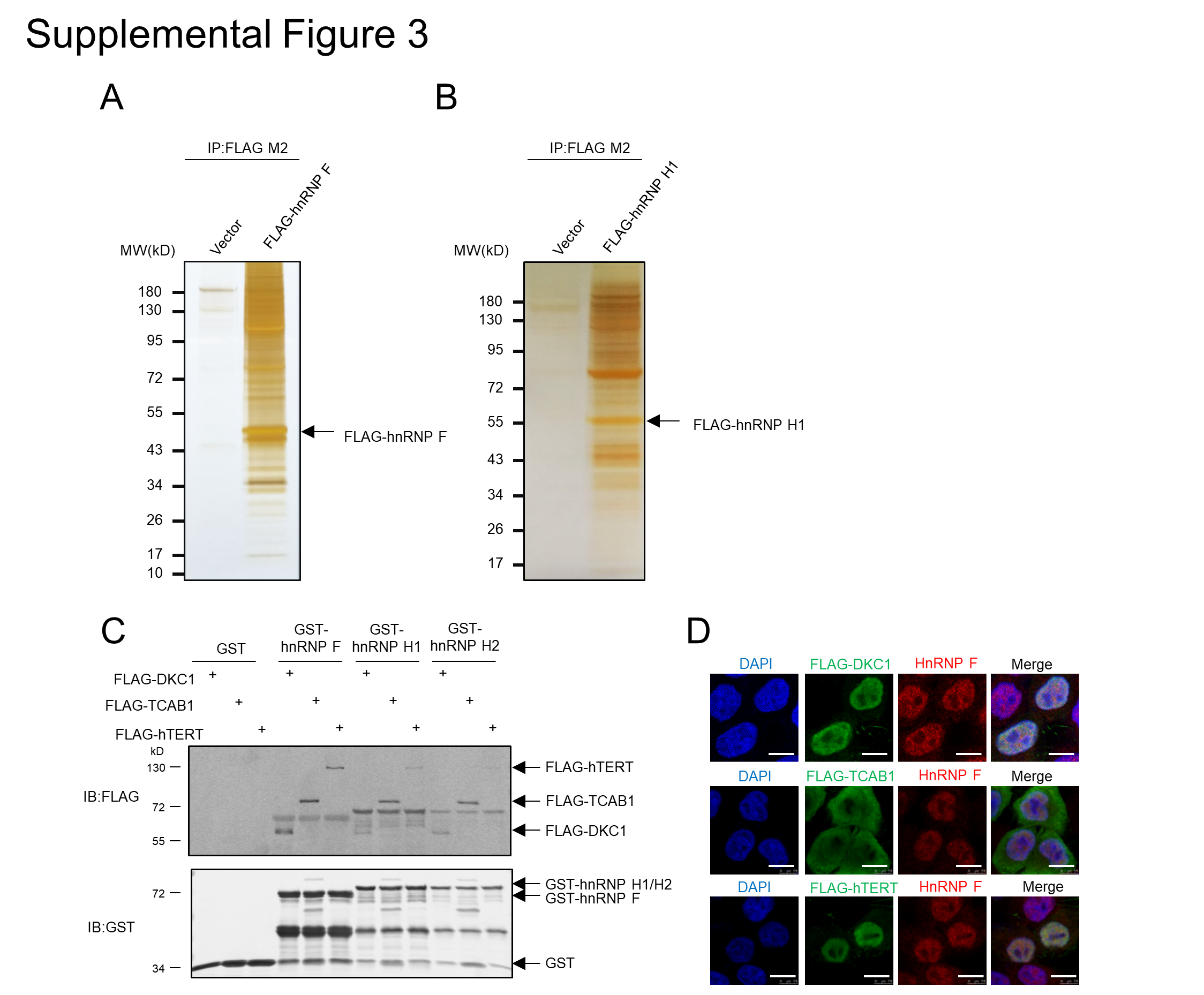

Supplement: Supplementary file 4 — Supplemental Figure 3 [file 41418_2019_483_MOESM4_ESM.tif]

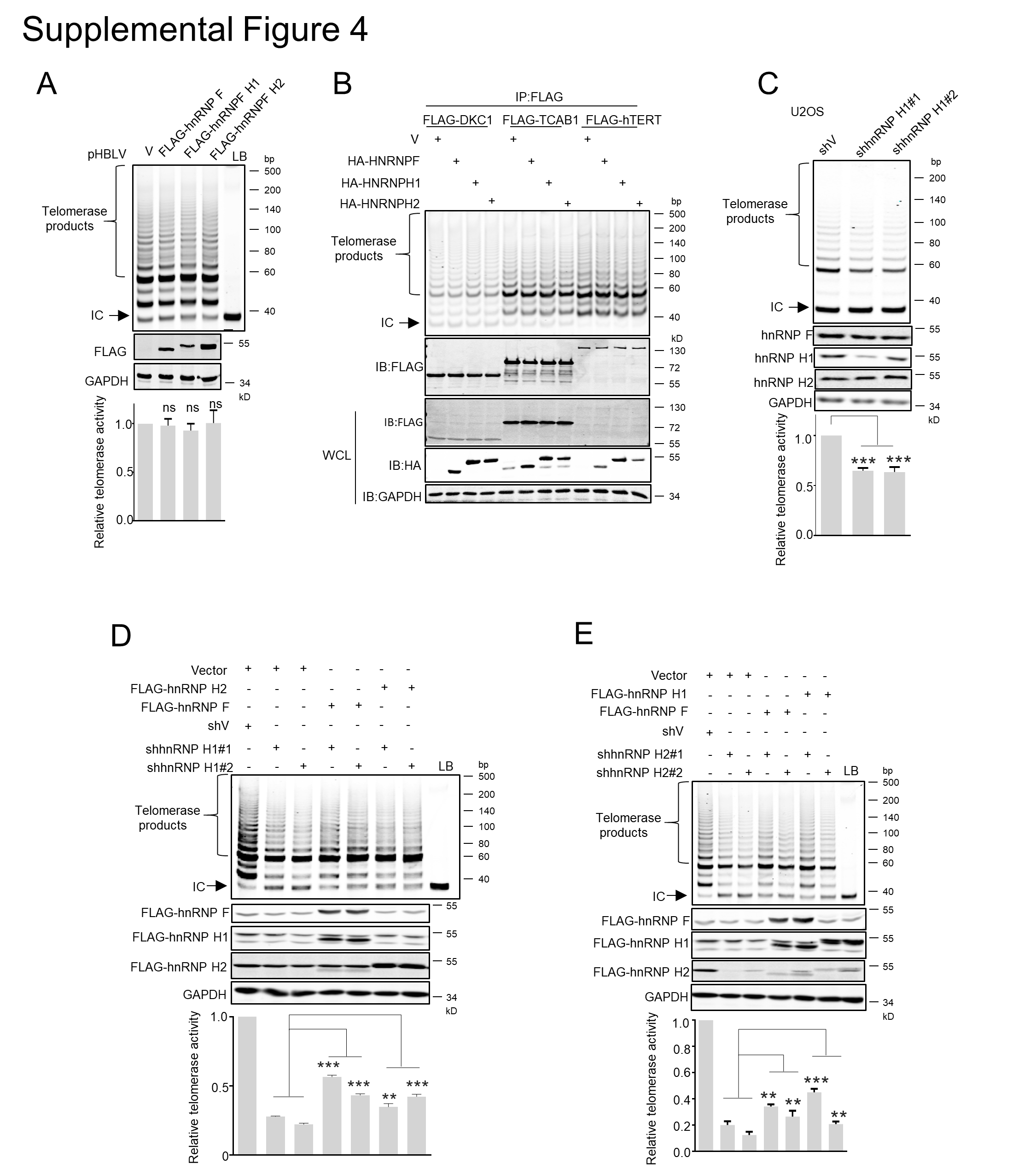

Supplement: Supplementary file 5 — Supplemental Figure 4 [file 41418_2019_483_MOESM5_ESM.tif]

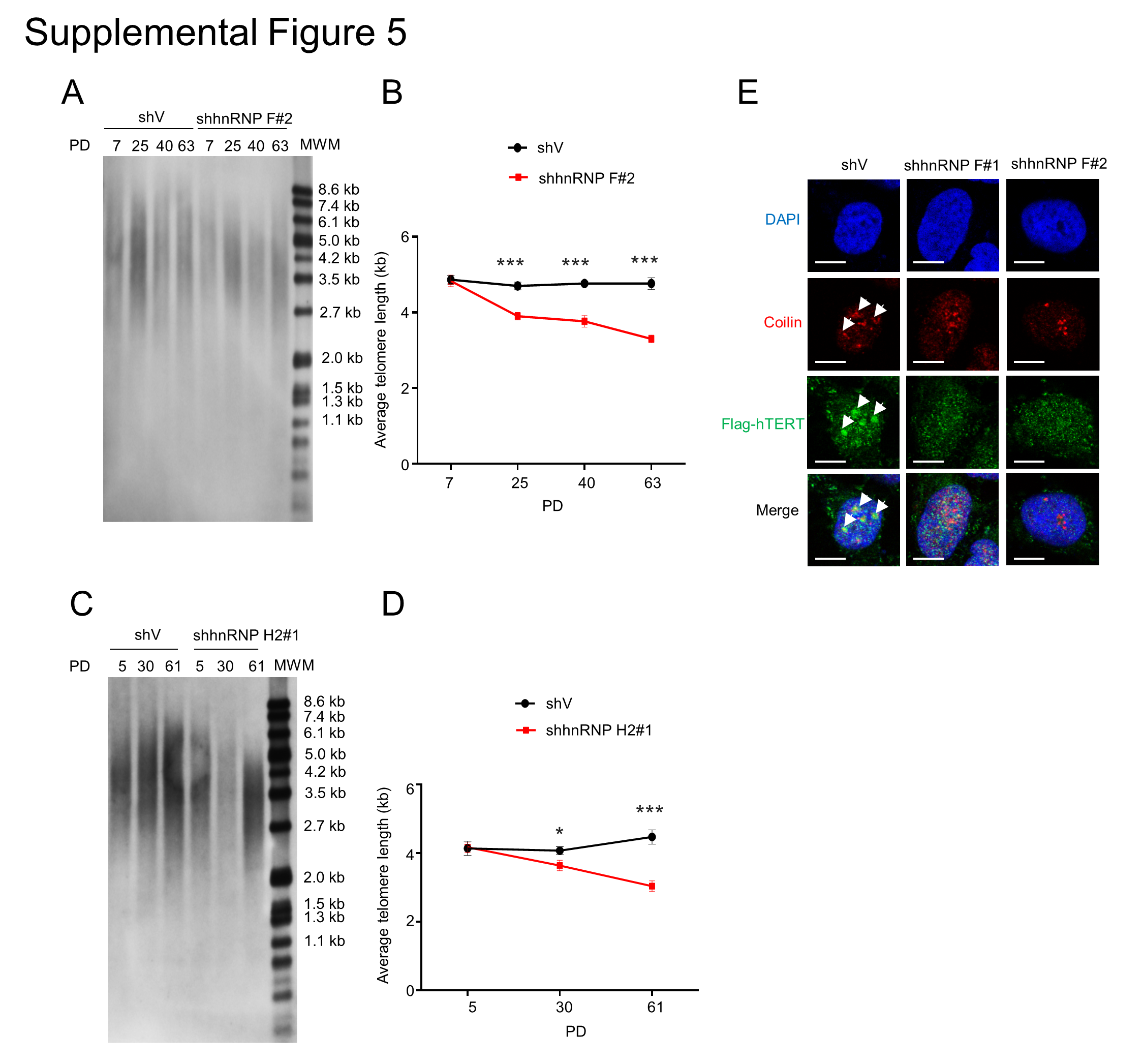

Supplement: Supplementary file 6 — Supplemental Figure 5 [file 41418_2019_483_MOESM6_ESM.tif]

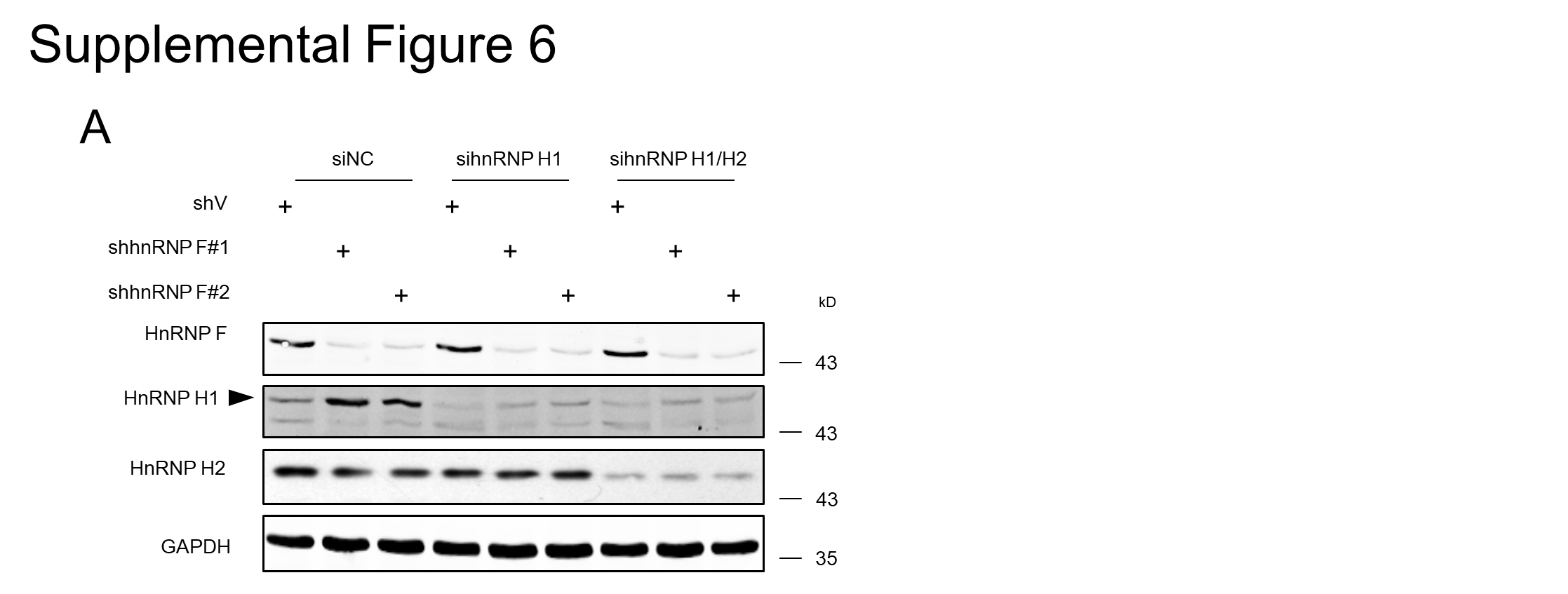

Supplement: Supplementary file 7 — Supplemental Figure 6 [file 41418_2019_483_MOESM7_ESM.tif]

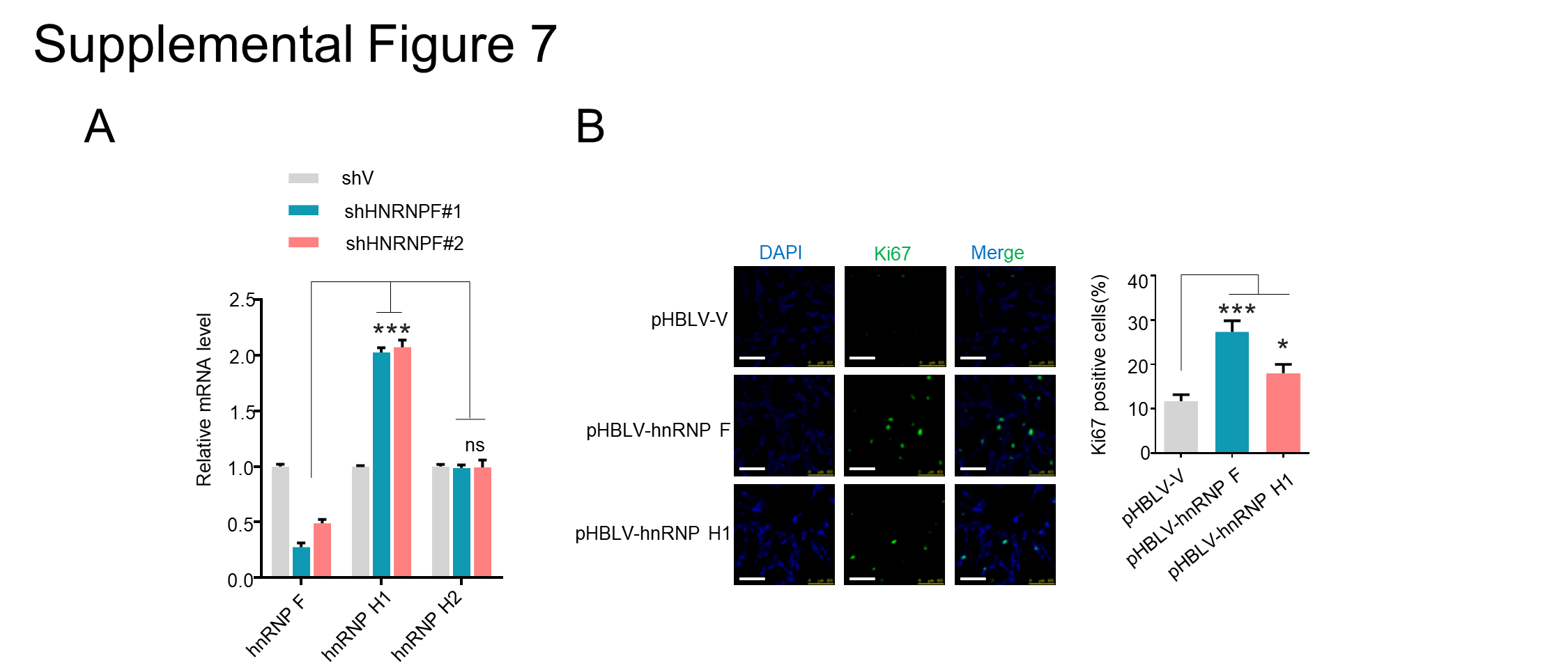

Supplement: Supplementary file 8 — Supplemental Figure 7 [file 41418_2019_483_MOESM8_ESM.tif]
